# Significant nutritional variables in patients with eating disorders

**DOI:** 10.1186/2050-2974-3-S1-O62

**Published:** 2015-11-23

**Authors:** Leanne Barron, Robert Barron, Jeremy Johnson, Shannon Ward, Ingrid Wagner, Warren Ward

**Affiliations:** 1 Brisbane City Doctors, Brisbane, Australia; 2 QUT ED CLINIC, Brisbane, Australia; 3 Charles Sturt University, New South Wales, Australia; 4 University of Queensland Medical School, Brisbane, Australia; 5 University of Queensland, Brisbane, Australia; 6 QUT Eating Disorder Clinic, Brisbane, Australia; 7 Queensland Eating Disorders Outreach Service, Brisbane, Australia

## 

Retrospective chart analysis of 113 patients presenting to a general practitioner with eating disorders was conducted, in an attempt to identify statistically and clinically significant nutritional variables. Blood tests are a useful diagnostic tool in eating disorders, and this research suggests that current testing should be broadened to include trace minerals such as zinc and manganese.

Results were analysed for cholesterol, red blood cell folate, vitamin B12, magnesium, manganese, zinc, vitamin D, phosphate, ferritin, white cell count, red cell count and platelets. Patients were analysed as an entire group, but also separately as those suffering from Anorexia Nervosa (1), Bulimia Nervosa (2), EDNOS (3) and classic AN followed by BN (4).

Analysis using T tests and chi squared showed that variables most likely to lie outside the population reference range were manganese, cholesterol, ferritin, vitamin B12, zinc and vitamin D.

